# Supplementary material for: Mechanisms governing the pioneering and redistribution capabilities of the non-classical pioneer PU.1
Source: Nat Commun. 2020 Jan 21;11:402. doi: 10.1038/s41467-019-13960-2 (PMC6972792; doi:10.1038/s41467-019-13960-2)
Supplement: Supplementary file 7 — Source data [file 41467_2019_13960_MOESM7_ESM.zip › Source_Data/FigureS2/FigureS2B_MotifScanOutput/KmeansCluster7/homerResults/motif4.similar.html]

motif4

## Information for motif4

G
T
A
C
G
C
T
A
A
G
T
C
G
A
C
T
G
A
C
T
G
A
T
C
G
A
T
C
G
T
A
C
G
A
T
C
G
T
A
C
G
C
A
T
A
G
C
T
G
A
C
T
  
Reverse Opposite:  

C
T
G
A
T
C
G
A
C
G
T
A
C
A
T
G
C
T
A
G
C
A
T
G
C
T
A
G
C
T
A
G
C
T
G
A
C
T
G
A
T
C
A
G
C
G
A
T
C
A
T
G
  

|  |  |
| --- | --- |
| p-value: | 1e-406 |
| log p-value: | -9.364e+02 |
| Information Content per bp: | 1.615 |
| Number of Target Sequences with motif | 1176.0 |
| Percentage of Target Sequences with motif | 36.64% |
| Number of Background Sequences with motif | 3433.2 |
| Percentage of Background Sequences with motif | 7.85% |
| Average Position of motif in Targets | 109.0 +/- 50.8bp |
| Average Position of motif in Background | 99.4 +/- 76.6bp |
| Strand Bias (log2 ratio + to - strand density) | 0.7 |
| Multiplicity (# of sites on avg that occur together) | 1.03 |
| Motif File: | file (matrix) reverse opposite |

### Similar de novo motifs found

|  |  |  |  |  |  |  |  |
| --- | --- | --- | --- | --- | --- | --- | --- |
| Rank | Match Score | Redundant Motif | P-value | log P-value | % of Targets | % of Background | Motif file |
| 1 | 0.868 | A T G C G T C A A G T C A G C T A C G T A G T C A G T C G A T C G C A T A T G C A G C T A C G T A T G C | 1e-242 | -557.563570 | 15.17% | 1.74% | motif file (matrix) |
| 2 | 0.952 | G T A C G C T A A T G C G A C T G A C T A G T C G A T C G A T C G A T C G T A C G C A T A G C T C G A T A T C G | 1e-165 | -381.038905 | 13.12% | 2.12% | motif file (matrix) |
| 3 | 0.826 | A G T C G C T A A G T C A G C T C G A T G T A C A G T C G T A C G A T C A G T C A C G T C A G T A G T C C G T A | 1e-26 | -60.247385 | 1.06% | 0.04% | motif file (matrix) |
